# The Problem of the Presence of DNA in Cosmetic and Medicinal Products Obtained from Animals on the CITES List

**DOI:** 10.3390/genes16070805

**Published:** 2025-07-08

**Authors:** Aleksandra Figura, Magdalena Gryzinska, Andrzej Jakubczak

**Affiliations:** Institute of Biological Basis of Animal Production, University of Life Sciences in Lublin, 20-950 Lublin, Poland; aleksandra.figura@up.lublin.pl (A.F.); andrzej.jakubczak@up.lublin.pl (A.J.)

**Keywords:** DNA analysis, species identification, smuggling of endangered species, pharmaceutical products, cosmetic product, brown bear, medicinal leech

## Abstract

Background: The illegal trade in wildlife remains a critical threat to biodiversity, prompting the development of international regulatory frameworks such as the Convention on International Trade in Endangered Species of Wild Fauna and Flora (CITES). One of the key challenges in enforcement is the detection of CITES-listed species in highly processed consumer goods. Methods: This study investigates the use of molecular techniques to detect animal DNA in two selected commercially available medicinal products—a balm and a gel—marketed with ingredients suggestive of protected species such as the brown bear (*Ursus arctos*) and the medicinal leech (*Hirudo medicinalis*). Results: Although DNA from these target species was not detected, the analysis revealed the presence of genetic material from the American mink (*Neovison vison*) and domestic pig (*Sus scrofa*), indicating the undeclared use of animal-derived substances. While limited in scope, these findings suggest potential ethical and transparency concerns, particularly for consumers adhering to vegetarian, vegan, or religious dietary practices. Conclusions: The study illustrates the feasibility of applying DNA-based screening methods in complex, degraded matrices and their potential as supportive tools in consumer product assessment. However, broader studies are necessary before drawing general regulatory or conservation conclusions.

## 1. Introduction

The illegal trade in endangered species remains a major global threat to biodiversity. Among the key instruments designed to counteract this phenomenon is the Convention on International Trade in Endangered Species of Wild Fauna and Flora (CITES), which regulates international trade in protected species. The European Union has implemented CITES provisions through a series of legal acts, including Council Regulation (EC) No. 338/97 and Commission Regulations (EU) No. 865/2006, No. 750/2013, and No. 578/2013 [1,2,3,4,5]. Since joining the European Union, Poland has consistently ranked among the leading countries in CITES-related border controls and seizures. This is largely due to its strategic location between Eastern Europe, Asia, and Western Europe, as well as the effectiveness of Polish customs in detecting illegal wildlife products. Between 2013 and 2015, over 24,000 specimens of CITES-listed fauna and flora were intercepted at Polish borders, with earlier years—such as 2008 and 2014—showing even higher figures, particularly in traditional Asian medicines containing ingredients from protected species like bears, seahorses, and leeches [6]. Globally, the illegal wildlife trade is estimated by the World Wildlife Fund (WWF) to be worth around USD 19 billion annually, making it one of the most profitable black-market industries, following drug, arms, and human trafficking [7]. Products derived from endangered species are often disguised as traditional or natural remedies, complicating both their detection and regulatory enforcement. These products, frequently processed into balms, gels, or powders, may appear harmless to consumers while actually containing components from protected species. Although some studies have examined the microbiological safety of cosmetics, there is a noticeable lack of research concerning the presence of undeclared animal DNA in such preparations [8]. This gap is particularly relevant in the context of global wildlife trade and biopiracy, which continue to pose a major threat to biodiversity and present significant challenges for existing enforcement mechanisms [9]. Of special concern are materials derived from species such as the brown bear (*Ursus arctos*) and the medicinal leech (*Hirudo medicinalis*), which are commonly used in folk medicine and traditional therapies, despite their legal protection under CITES [10]. Bear fat is traditionally believed to have anti-inflammatory and analgesic properties, and is often used in balms intended for the treatment of joint pain, muscle stiffness, and respiratory conditions such as bronchitis [11]. Similarly, extracts from the medicinal leech, particularly hirudin—a powerful anticoagulant—are incorporated into gels and creams marketed for the treatment of varicose veins, circulatory disorders, and hematomas [12].

Molecular biology techniques, particularly those based on mitochondrial DNA (mtDNA), offer valuable tools for species identification, even in highly processed or degraded samples [7]. The *cytochrome b* gene is widely used for identifying vertebrate species, while other mtDNA markers such as *COI* and the D-loop region provide additional resolution, particularly for closely related taxa. These methods are increasingly applied in wildlife forensics, yet their use in the analysis of cosmetics and medicinal products remains limited [7,13,14,15].

However, despite their broad applicability, these mtDNA-based approaches also come with certain limitations that must be acknowledged. One important limitation of *cytochrome b* sequencing lies in its species resolution. While the mitochondrial *cytochrome b* gene is commonly used for species identification due to its variability, it may not always distinguish between closely related species, especially those within the same genus. This could lead to ambiguous or inconclusive results in cases where high taxonomic precision is required [16,17].

Additionally, the risk of false negatives may arise from DNA degradation, especially in highly processed or aged samples, which can hinder successful amplification. On the other hand, false positives may result from contamination during sampling, extraction, or PCR. Therefore, proper controls, laboratory precautions, and replication are essential to minimize these risks [18,19,20]. Integrating mitochondrial data with nuclear DNA markers can improve species-level identification and help clarify mitochondrial–nuclear incongruence, which has been documented in many animal systems and may obscure phylogenetic or biogeographic patterns [21].

The present study addresses this gap by applying molecular methods targeting the *cytochrome b* gene to investigate the presence of DNA from CITES-listed species in two commercially available products—a balm and a gel—marketed as containing animal-derived ingredients. The aim was to determine whether these products contained genetic material from the brown bear (*U. arctos*) or the medicinal leech (*H. medicinalis*), and to assess the feasibility of using genetic tools in the context of a small-scale analysis of complex consumer products.

## 2. Materials and Methods

The material for the study consisted of two medicinal products, a gel and a balm, secured during proceedings at the Polish border crossing. Radiculin Walentina Dikula (RWD) gel (100 mL) and Ven Activ Forte (VAF) balm (125 mL) were in sealed plastic tubes and packaged in their original cardboard packaging, containing information in Russian and Polish. Active substances and ingredients in the tested products declared by the manufacturer are included in Table 1.

DNA was isolated from the cosmetic and medicinal gel and balm using the Sherlock AX kit (A&A Biotechnology, Gdansk, Poland). PCR was carried out using universal mtDNA (*cytochrome b*) primers intended for species identification, i.e., L14841 (F: AAAAAGCTTCCATCCAACATCTCAGCATGATGAAA) and H15149 (R: AAACTGCAGCCCCTCAGAATGATATTTGTCCTCA) [22]. The composition of the PCR reaction mixture was as follows: 50 ng DNA, 2 μL of dNTP (2.5 mM each of dATP, dCTP, dGTP, and dTTP), 2.75 μL of 10× PCR AmpliTaq 360 buffer, 2 μL of MgCl_2_ (25 mM), 1.2 μL of 360GC Enhancer, 0.12 μL of AmpliTaq Gold 360 polymerase (Applied Biosystems, Foster City, CA, USA) and 0.2 μL of each primer (F and R). The mixture was made up to 20 μL with sterile, deionized water. The following temperature/time profile was used: initial denaturation at 95 °C—7 min; 35 cycles—denaturation (95 °C—45 s); primer annealing (53 °C—45 s); elongation (72 °C—45 s); and final elongation—7 min. Negative controls, consisting of PCR reactions without DNA template, were used to rule out any potential contamination of the PCR reagents or samples. Due to the limitations in obtaining authentic samples of bear or leech DNA for direct validation, the study’s validation process was limited to bioinformatic comparisons with known reference sequences in the NCBI database. The PCR product was purified using the EPPiC Fast kit (A&A Biotechnology). Sequencing PCR (bidirectional DNA sequencing) was carried out using the DNA BigDye^®^ Terminator v3.1 (Applied Biosystems) procedure, and then the terminators were removed using the ExTerminator kit (A&A Biotechnology). Sequencing reactions were carried out in the 3100-Avant Genetic Analyzer 4-Capillary Array (Applied Biosystems). The results were analyzed using GeneMapper^®^ ID-X v.1.2 (Applied Biosystems). The nucleotide sequence of the fragment of cytochrome b mtDNA was compared in the NCBI (National Center for Biotechnology Information, Bethesda, MD, USA) database using BLAST (Basic Local Alignment Search Tool, version 2.16.0).

## 3. Results

The use of a pair of universal primers made it possible to obtain a PCR product of a fragment of the cytochrome b gene for the test samples.

Table 1 and Table 2 present the animal species determined based on sequencing of the mtDNA fragment for the gel and balm. In the case of the gel, the fragment of cytochrome b shows varying degrees of coverage with 17 animal species. The closest similarity of the DNA sequences of the fragment is to American mink (*Neovison vison*), at a level of 97.61–99.17%, and the degree of coverage is 68–83%. The similarity of the DNA sequence to the Colombian weasel (*Mustela felipei*) and long-tailed weasel (*Mustela frenata*) is 90.41%, and the degree of coverage is 82%. The similarity of the DNA sequence is lower for the Amazon weasel (*Mustela africana*), at 90.07%, with 82% coverage. The similarity of the DNA sequence to other species, i.e., back-striped weasel (*Mustela strigidorsa*), mountain weasel (*Mustela altaica*), Asian badger (*Meles leucurus*), Malayan weasel (*Mustela nudipes*), hairy-nosed otter (*Lutra sumatrana*), European badger (*Meles meles*), Japanese badger (*Meles anakuma*), Western spotted skunk (*Spilogale gracilis*), pygmy spotted skunk (*Spilogale pygmaea*), stoat (*Mustela erminea*), greater hog badger (*Arctonyx collaris*), Eurasian otter (*Lutra lutra*), and yellow-bellied weasel (*Mustela kathiah*), ranged from 89.76% to 86.64%. The degree of coverage of the fragment for these species ranged from 83% to 81%. Of the species named above, five are protected by CITES (Table 2). In the case of the balm (Table 3), an 81.20–81.67% similarity of the DNA of the sequence with wild boar/domestic pig was obtained.

**Figure 1:**
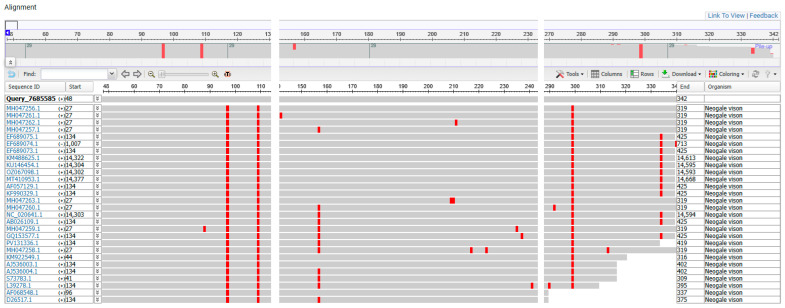
MSA Viewer multiple nucleotide sequence alignment report indicating *Neovison vison*.

**Figure 2:**
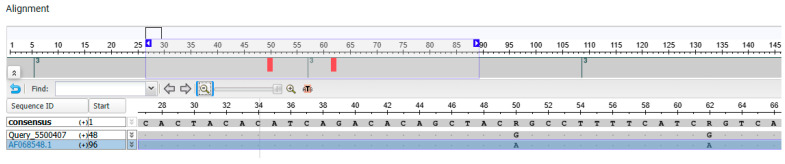
DNA sequence indicating the species American mink (*Neovison vison*) according to the NCBI database (GenBank: AF068548.1).

The balm was found to contain biological material (fat) from only one species, the pig, while 17 species were detected in the gel. The MSA (multiple sequence alignment) Viewer was used to generate a report using all sequences enabling significant alignments (Figure 1). On this basis the closest DNA sequence deposited in the NCBI database to that obtained for the sample (gel) was found to be GenBank: AF068548.1 (Figure 2).

## 4. Discussion

Due to increased border controls following Poland’s accession to the European Union and the improved monitoring of intercepted contraband, the scale of illegal trade in endangered animal and plant species has become more apparent. The majority of illegally transported animals are sea anemones and corals (*Anthozoa*) and turtles (*Testudines*), as well as medicinal leeches (*H. medicinalis*) and bivalves of the order *Veneroida* spp. [23]. Half of the intercepted specimens belong to species used in traditional Chinese medicine (TCM) [24].

Although our analysis did not detect brown bear DNA, this species has previously been subject to numerous CITES-related seizures [25]. Between 1999 and 2011, the Polish Customs Service intercepted bear skins and a paw fragment, and products containing bear bile or fat continue to be smuggled [24,26]. In TCM, bear bile is valued for its medicinal properties and used in products like Xiong Dan [23,24]. The acquisition of bear bile, commonly used in traditional medicine, typically involves keeping the animals in confined cages and extracting bile through surgically implanted catheters. This procedure has been described as highly invasive and raises significant animal welfare and ethical concerns [27,28]. Confiscated items have included hundreds of packages of creams and medicines containing bear-derived substances, both in Poland and globally. For example, Interpol’s Operation TRAM in 2010 led to worldwide seizures of TCM products containing derivatives from bears, tigers, and rhinoceroses, with an estimated total value of EUR 10 million [29].

Similarly, although medicinal leech DNA was not detected in the analyzed samples, past customs data confirm their frequent illegal trade. Extracts from *H. medicinalis*, still used in folk and alternative medicine for their bioactive compounds, are present in various topical preparations [26]. Due to their single-use application in hirudotherapy, the demand for live leeches remains high [23]. In 2009, 800 leeches were intercepted, followed by a record 8070 in 2013 and 1036 in 2014 [6,24]. Processed products also continue to be seized, including 2100 packages in 2011 and 77 packages of leg balm in 2014 [24]. In 2015, Operation Cobra III by Interpol and Europol resulted in the arrest of 139 individuals and the interception of various wildlife products, including medicinal leeches, ivory, and frozen eel [29].

Species identification methods have primarily been used for detecting livestock species [7,30]. The *cytochrome b* gene fragment allows for precise differentiation among eukaryotic organisms. In this study, genetic material with 98% similarity to American mink was identified. According to Kiewlicz (2013), mink fat is increasingly used in skin and hair care products for its beneficial properties [31,32]. As early as 1995, it appeared in 139 cosmetic items, including shampoos, conditioners, bath gels, lip balms, sunscreens, and moisturizers. Cosmetic Ingredient Review (CIR) data from the same year showed mink fat in 2% of sun creams and 0.2% of hair products [33].

Pig DNA was also detected in the samples, confirming the presence of pork derivatives in cosmetics. Historically, pork fat (lard or fatback) was widely used in folk medicine to treat skin conditions, infections, and digestive issues [34]. Currently, pork fat is used in the production of ointments. Fat from the abdominal cavity of pigs is melted in a water bath and then filtered while still hot. Lard is highly pure and easily absorbed, though it has a short shelf life and turns rancid quickly. Despite this, it remains an ingredient in certain creams. Its resistance to washing off makes it an effective protective and softening layer. Additionally, it rarely causes allergic reactions, which is beneficial for individuals with problematic skin (e.g., psoriatic or atopic), inflammation, or fungal infections, such as when used in ointments containing potassium iodide [35].

The presence of pork derivatives in cosmetics raises significant religious concerns. In both Judaism and Islam, pork is strictly forbidden, and this prohibition extends to personal care products containing pig-derived ingredients. Common cosmetic substances such as collagen, glycerin, and fatty acids may come from non-halal or non-kosher sources [36]. The concepts of halal/kosher (permitted) and haram/treyf (forbidden) are essential in determining ingredient acceptability, reflecting not only spiritual beliefs but also concerns about hygiene and potential health risks [37,38,39].

For compliance, both the ingredients and production processes must adhere to specific religious standards [40]. Since cosmetics can be absorbed through the skin, ingested (e.g., lipstick), or inhaled (e.g., perfume), the importance of halal/kosher certification is growing [41]. Likewise, vegans and vegetarians avoid animal-derived ingredients for ethical or health reasons, favoring plant-based alternatives. Accurate labeling is thus essential—not only for religious and ethical adherence but also for consumer autonomy.

From a regulatory perspective, mislabeling is also a key issue. Regulation (EU) No 1169/2011 on the provision of food information to consumers includes provisions relevant to cosmetic and medicinal products, ensuring transparency regarding product composition [42]. Directive 2001/95/EC on general product safety further requires accurate labeling and prohibits misleading information about ingredient origin or composition. Failure to comply may mislead consumers, particularly those adhering to religious, vegan, or vegetarian standards. Correct labeling is thus crucial for consumer protection and legal compliance within the EU [43]. Stricter enforcement mechanisms may include the mandatory DNA screening of high-risk products, the enhanced documentation of raw material sourcing, and stricter penalties for non-compliance. Regulatory agencies could also establish certification programs for “animal-free” or “accurately labeled” products, similar to halal, kosher, or vegan labeling systems. To enhance compliance, regulatory oversight could be supported by clearer labeling standards, the improved traceability of raw materials, and more frequent product audits. Encouraging transparency and accountability within the supply chain may serve as a deterrent against mislabeling practices [44].

Notably, a survey of law enforcement professionals ranked genetic testing as the second most valuable forensic tool after fingerprint analysis, while judges and prosecutors considered it the most probative form of evidence [45]. This underscores the potential of DNA-based species identification methods, which, as demonstrated in this case study, can be successfully applied even to highly processed products [7]. Although the results are promising, further validation through large-scale studies involving more diverse sample types is needed.

## 5. Conclusions

This case study confirms the applicability of molecular genetic techniques for detecting undeclared animal-derived components in complex, highly processed consumer products. The identification of genetic material from *Sus scrofa* (pig) and *Neovison vison* (American mink) in products lacking such label declarations raises concerns about ingredient transparency and labeling accuracy. In contrast, the absence of DNA from *Ursus arctos* (brown bear) and *Hirudo medicinalis* (medicinal leech) underscores the need for the empirical verification of product claims.

Given the preliminary nature and limited sample size of this study, the results do not support broad regulatory or conservation conclusions. However, they highlight the potential of DNA-based methods as supportive tools for consumer protection, improved transparency, and enforcement against mislabeling or the illegal use of animal-derived substances. Incorporating such molecular approaches into routine product monitoring could enhance accountability in the cosmetic and medicinal product sectors.

## Figures and Tables

**Table 1:** Active substances and ingredients in the tested products declared by the manufacturer.

	Radiculin Walentina Dikula (RWD) Gel	Ven Activ Forte (VAF) Balm
Active substances	bear bile, bee venom, mumio, extracts and oils from medicinal plants	medical leech extract, troxerutin, horse chestnut, extracts and oils from medicinal plants
Ingredients	water, liposome emulsion complex no. 3: (stearic acid, emulsion wax Neowax, glycerin, vegetable oil, anhydrous lanolin, propylene glycol, microcar IT, nimesulide, sodium hydroxide, Grindox antioxidant), hemp oil, juniper essential oil, elecampane extract, cinquefoil extract, pepper extract, pine bud extract, St. John’s wort extract, thyme extract, angelica extract, mumio, peavit, bee venom, bear bile, vitanol, collagen hydrolyzate, D-panthenol, microcar DMP	water, glycerin, carbomer, cococaprilate, dicaprylyl ether, troxerutin, grape extract, horse chestnut extract, collagen hydrolyzate, chamomile oil extract, sophora japonica, horsetail, rosehip fruit, propylene glycol extract, aloe vera, arnica, birch, ginkgo biloba, green tea, badiaga, D-panthenol, cottonseed oil, hazelnut oil, sea buckthorn oil, leech extract, vitanol, tea tree oil, lemon oil, phenoxyethanol, ethylhexylglycerin, sodium hydroxide, disodium EDTA, limonene

**Table 2:** Animal species determined based on sequencing of *cytochrome b* in Radiculin Walentina Dikula gel.

	DNA Sequence Similarity (%)	Max Score	E-Value	Degree of Coverage of Fragment (%)	Species—Common Name	Species—Latin Name
1.	97.61–99.17	431–525	1 × 10^−142^	68–83	American mink	*Neovison vison* formerly *Mustela vison*
2.	90.41	385	1 × 10^−102^	82	Colombian weasel	*Mustela felipei*
3.	90.41	385	1 × 10^−102^	82	Long-tailed weasel	*Mustela frenata*
4.	90.07	379	7 × 10^−101^	82	Amazon weasel	*Mustela africana*
5.	89.42–89.76	368–374	3 × 10^−99^	82	Back-striped weasel	*Mustela strigidorsa*
6.	87.71–88.05	340–346	7 × 10^−91^	82	Mountain weasel *	*Mustela altaica*
7.	87.59	337	4 × 10^−88^	82	Asian badger	*Meles leucurus*
8.	87.46	339	1 × 10^−88^	83	Malayan weasel	*Mustela nudipes*
9.	87.33	335	1 × 10^−87^	82	Hairy-nosed otter *	*Lutra sumatrana*
10.	86.90–87.59	326–337	2 × 10^−86^	82	European badger	*Meles meles*
11.	86.90–87.24	326–331	2 × 10^−86^	82	Japanese badger	*Meles anakuma*
12.	86.76–87.15	320–326	4 × 10^−83^	81	Western spotted skunk	*Spilogale gracilis*
13.	86.76	320	4 × 10^−83^	81	Pygmy spotted skunk	*Spilogale pygmaea*
14.	86.64–87.03	322–329	1 × 10^−83^	82	Stoat *	*Mustela erminea*
15.	86.64	324	3 × 10^−84^	82	Greater hog badger	*Arctonyx collaris*
16.	86.64	324	3 × 10^−84^	82	Eurasian otter *	*Lutra lutra*
17.	86.64	324	3 × 10^−84^	82	Yellow-bellied weasel *	*Mustela kathiah*

* Species on CITES list [1].

**Table 3:** Animal species determined based on sequencing of cytochrome b in Radiculin Ven Activ Forte balm.

	DNA Sequence Similarity (%)	Max Score	E-Value	Degree of Coverage of Fragment (%)	Species—Common Name	Species—Latin Name
1.	81.20–81.67	259–265	2 × 10^−66^	93–95	Wild boar/domestic pig	*Sus scrofa*

## Data Availability

Data are contained within the article.
